# ATP-Binding Cassette Protein ABCC10 Deficiency Prevents Diet-Induced Obesity but Not Atherosclerosis in Mice

**DOI:** 10.3390/ijms232213813

**Published:** 2022-11-10

**Authors:** Abeer Al Otaibi, Sindiyan Alshaikh Mubarak, Ali Al Qarni, Abbas Hawwari, Ahmed Bakillah, Jahangir Iqbal

**Affiliations:** King Abdullah International Medical Research Center-Eastern Region, King Abdulaziz Hospital, King Saud Bin Abdulaziz University for Health Sciences, Ministry of National Guard Health Affairs, Al Ahsa 31982, Saudi Arabia

**Keywords:** diet-induced obesity, type 2 diabetes, atherosclerosis, lipid metabolism, triglyceride absorption

## Abstract

Excess plasma lipid levels are a risk factor for various cardiometabolic disorders. Studies have shown that improving dyslipidemia lowers the progression of these disorders. In this study, we investigated the role of ATP-binding cassette transporter C10 (ABCC10) in regulating lipid metabolism. Our data indicate that deletion of the *Abcc10* gene in male mice results in lower plasma and intestinal triglycerides by around 38% and 36%, respectively. Furthermore, deletion of ABCC10 ameliorates diet-induced obesity in mice and leads to a better response during insulin and glucose tolerance tests. Unexpectedly, ABCC10 deficiency does not affect triglyceride levels or atherosclerosis in ApoE-deficient mice. In addition, our studies demonstrate low oleate uptake by enterocytes (~25–30%) and less absorption (~37%) of triglycerides in the small intestine of ABCC10 knockout mice. Deletion of the *Abcc10* gene also alters several lipid metabolism genes in the intestine, suggesting that ABCC10 regulates dietary fat absorption, which may contribute to diet-induced obesity in mice.

## 1. Introduction

Lipids play a critical role in biological life and are important components in the cell structure. They are involved in several metabolic and hormonal pathways. Dietary and endogenous lipids are transported in plasma by lipoproteins that are synthesized and secreted by enterocytes and hepatocytes [1,2]. Hyperlipidemias could arise due to overproduction, decreased hydrolysis, or the impaired removal of apoB lipoproteins [3]. Numerous clinical trials and outcome studies have demonstrated that improving dyslipidemia lowers the progression of atherosclerosis, as well as the resulting adverse cardiovascular events [4]. Reducing plasma lipids using statins lowers the incidence of some of these disorders in only 20–40% individuals [5]. Therefore, there is a need to formulate new approaches or regimens to treat hyperlipidemia. It is possible that studies related to lipoprotein production and catabolism might provide new avenues to control plasma lipids.

The increased prevalence of obesity and sedentary life habits have given rise to an increased incidence of insulin resistance and type 2 diabetes, accompanied by dyslipidemia and an increased risk for cardiovascular diseases [6]. Similar lipid abnormalities, which mainly originate from the hepatic overproduction of very-low-density lipoproteins (VLDL) [7,8], are observed in insulin resistance patients with and without type 2 diabetes [9]. In type 2 diabetes patients, an increased plasma residence time of lipoproteins due to decreased liver uptake contributes to the augmented accumulation of triglycerides in plasma [10]. Furthermore, insulin resistance in these patients contributes to the increased production of chylomicrons, mainly due to the increased synthesis and secretion of apolipoprotein B48 (apoB48)-containing lipids and lipoproteins by the intestines [11].

To date, a total of 49 different ATP-binding cassette (ABC) transporters with diversified functions ranging from ion transport to macromolecular efflux have been identified in humans [12,13,14,15]. ABCA1 is well known to transport free cholesterol and phospholipids across the cell membrane [16,17]. We recently showed that ABCA1 is a critical determinant of plasma glycosphingolipids [18]. We have also shown that ABCA7 plays a role in the biosynthesis and efflux of sphingomyelin [19]. ABCC1 and ABCA12 have been shown to transport glucosylceramides in vitro [20] and in keratinocytes [21]. Recently, Budani et al. [22] showed that multiple ABC transporters potentially act as glucosylceramide flippases and differentially control glycosphingolipid biosynthesis. ATP-binding cassette transporter C10 (ABCC10) is a member of the multi-drug resistance protein that translocates diverse substrates across membranes [23]. It belongs to subfamily C of the large family of 48 evolutionarily conserved ABC transporters [24,25,26] and is involved in multi-drug resistance in cancer cells by preventing the intracellular accumulation of certain antitumor drugs. The broad specificity of ABCC10 transporter includes antitumor drugs, such as taxanes, cytarabine, vinca alkaloids, and epithilone B [23]. It also transports modulators of the estrogen pathway, such as tamoxifen [23]. A bioinformatics analysis of genes expressed in samples of atherosclerotic lesions and control arteries without atherosclerotic lesions showed increased expression of ABCC10 [27], suggesting a role of ABCC10 in atherogenesis. In this study, we aimed to investigate the impact of ABCC10 deletion on lipids and lipoprotein metabolism in relation to obesity and atherosclerosis, with the understanding that this knowledge might help to devise new strategies to control hyperlipidemia.

## 2. Results

### 2.1. Deletion of ABCC10 in Chow-Fed Mice Decreases Plasma and Tissue Triglycerides

**Figure 1:**
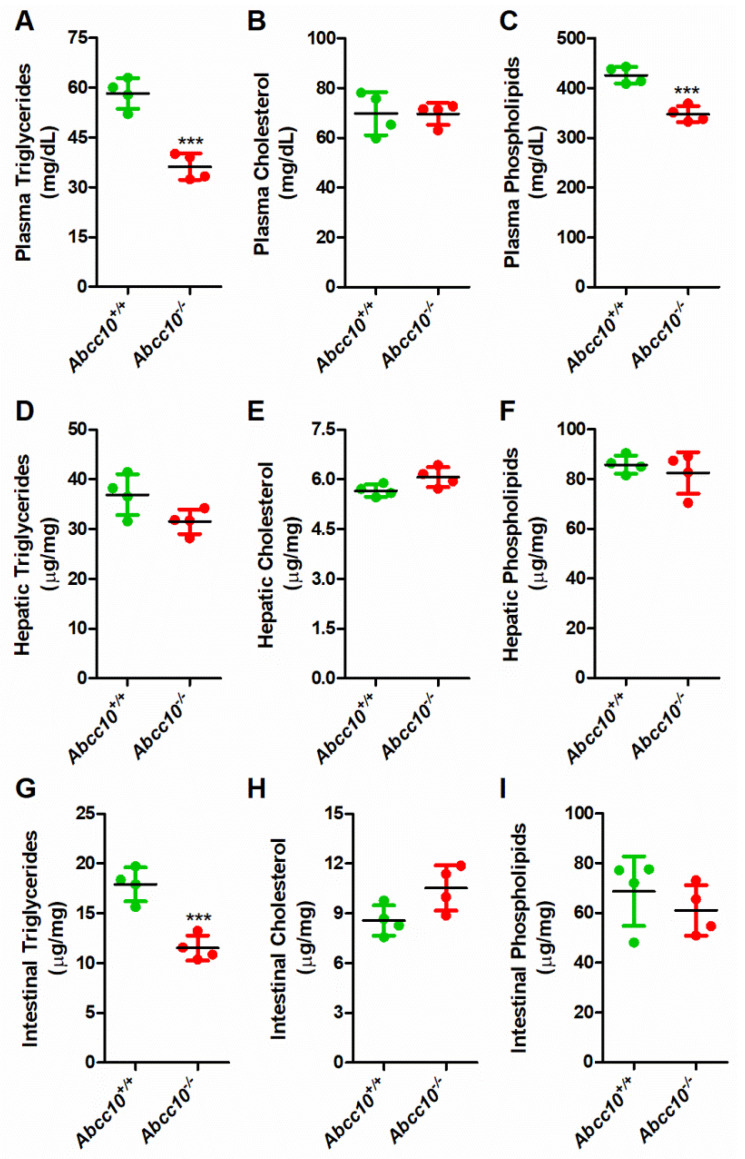
Deletion of *Abcc10* gene in mice decreases plasma and tissue triglycerides. Chow-diet-fed, 12-week-old *Abcc10^+/+^* and *Abcc10^−/−^* male mice (*n* = 4) were fasted overnight and triglycerides (**A**,**D**,**G**), cholesterol (**B**,**E**,**H**), and phospholipids (**C**,**F**,**I**) were measured in plasma (**A**–**C**), liver (**D**–**F**), and intestine (**G**–**I**). Values are plotted as replicates (mean ± SD). *** *p* < 0.001, as compared with *Abcc10^+/+^* mice.

To test whether ABCC10 plays a role in lipid metabolism, 12-week-old *Abcc10^+/+^* and *Abcc10^−/−^* mice fed a chow diet were used to measure plasma and tissue lipids (Figure 1). There was a significant decrease of 38% and 18% in the levels of triglycerides (Figure 1A) and phospholipids (Figure 1C), respectively, in the plasma of *Abcc10^−/−^* mice compared to chow-fed *Abcc10^+/+^* mice. We did not see any significant difference in the plasma cholesterol levels between *Abcc10^+/+^* and *Abcc10^−/−^* mice (Figure 1B). The liver and intestine are the main sources of lipids and lipid-containing lipoproteins in the plasma during the fed and fasting states. Next, we looked at the levels of lipids in these tissues. Lipids in the liver did not register any significant change (Figure 1D–F). However, there was a decreased trend in the hepatic triglycerides, but it was not statistically significant between the two groups (Figure 1D). Since we did not see any significant changes in the liver lipids, we hypothesized that the decrease in the plasma triglycerides may be due to the changes in the intestinal lipids. Again, cholesterol (Figure 1H) and phospholipids (Figure 1I) in the intestine were not changed. However, we saw a significant decrease in the levels of triglycerides (Figure 1G) by 36% in the intestines. These combined data suggest that the deletion of ABCC10 affects triglyceride metabolism in chow-diet-fed mice and might imply that the reduction in plasma triglycerides in ABCC10-deficient mice is influenced predominantly by changes in the intestinal lipids and not hepatic lipid levels.

### 2.2. Deletion of ABCC10 Prevents Diet-Induced Obesity in Mice

**Figure 2:**
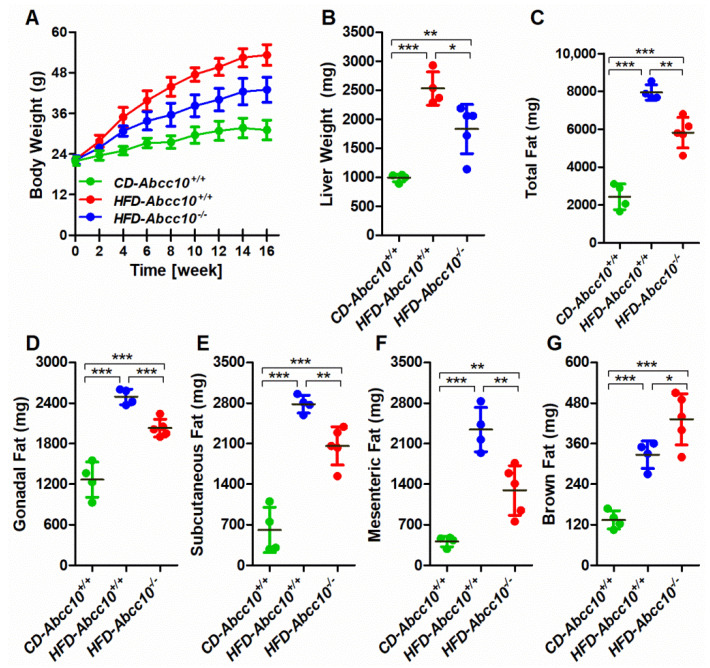
Ablation of ABCC10 in mice prevents diet-induced obesity. Eight-week-old *Abcc10^+/+^* (*HFD-Abcc10^+/+^*, *n* = 4) and *Abcc10^−/−^* (*HFD-Abcc10^−/−^*, *n* = 5) male mice were fed high-fat obesity diet for 16 weeks. As a control, age-matched *Abcc10^+/+^* (*CD-Abcc10^+/+^*, *n* = 4) male mice were kept on chow diet during this period. Body weight of animals was recorded every two weeks (**A**). At the end of the experiment, mice were fasted overnight and sacrificed. Liver weight (**B**), total fat (**C**), gonadal fat (**D**), sub-cutaneous fat (**E**), mesenteric fat (**F**), and brown fat (**G**) were measured. Values are plotted as replicates (mean ± SD). * p < 0.05, ** *p* < 0.01, and *** *p* < 0.001.

It is well established that increased intestinal lipid absorption is implicated in diet-induced obesity. Since we observed a significant decrease in the levels of plasma and intestinal triglycerides in chow-diet-fed *Abcc10^−/−^* mice compared to *Abcc10^+/+^* mice, we, therefore, looked at the effect of high-fat-diet feeding on lipid metabolism in these mice. We fed *Abcc10^+/+^* and *Abcc10^−/−^* mice with a high-fat diet for 16 weeks to induce obesity and measured the body weight every two weeks (Figure 2A). As a control, we also used chow-fed mice to assess the change in body weight. As expected, there was a significant increase in body weight in high-fat obesity-diet-fed *Abcc10^+/+^* (*HFD-Abcc10^+/+^*) mice over the period of 16 weeks as compared to chow-fed *Abcc10^+/+^* (*CD-Abcc10^+/+^*) mice (Figure 2A). Similarly, we observed an increase in the body weight in high-fat obesity-diet-fed *Abcc10^−/−^* (*HFD-Abcc10^−/−^*) mice compared to *CD-Abcc10^+/+^* mice, but this increase was lower than in the *HFD-Abcc10^+/+^* mice (Figure 2A). Increased body weight was associated with a significant increase in liver weight in *HFD-Abcc10^+/+^* mice compared to *CD-Abcc10^+/+^* mice, which was again lower in *HFD-Abcc10^−/−^* mice (Figure 2B).

Next, we measured the change in body fat in these mice. Similar to the increase in body weight, there was a significant increase in total body fat in *HFD-Abcc10^+/+^* mice compared to *CD-Abcc10^+/+^* mice, and this increase was significantly lower in *HFD-Abcc10^−/−^* mice (Figure 2C). The increase in total body fat in *HFD-Abcc10^+/+^* mice was due to an increase in gonadal fat (Figure 2D), subcutaneous fat (Figure 2E), mesenteric fat (Figure 2F), and brown fat (Figure 2G). However, there was a decrease in gonadal (Figure 2D), subcutaneous (Figure 2E), and mesenteric (Figure 2F) fat but an increase in the brown fat (Figure 2G) in *HFD-Abcc10^−/−^* mice. These data suggest that the deletion of ABBC10 prevents diet-induced obesity in mice.

### 2.3. Plasma Lipid and ApoB Lipoprotein Levels Are Lower in High-Fat-Diet-Fed ABCC10-Deficient Mice

**Figure 3:**
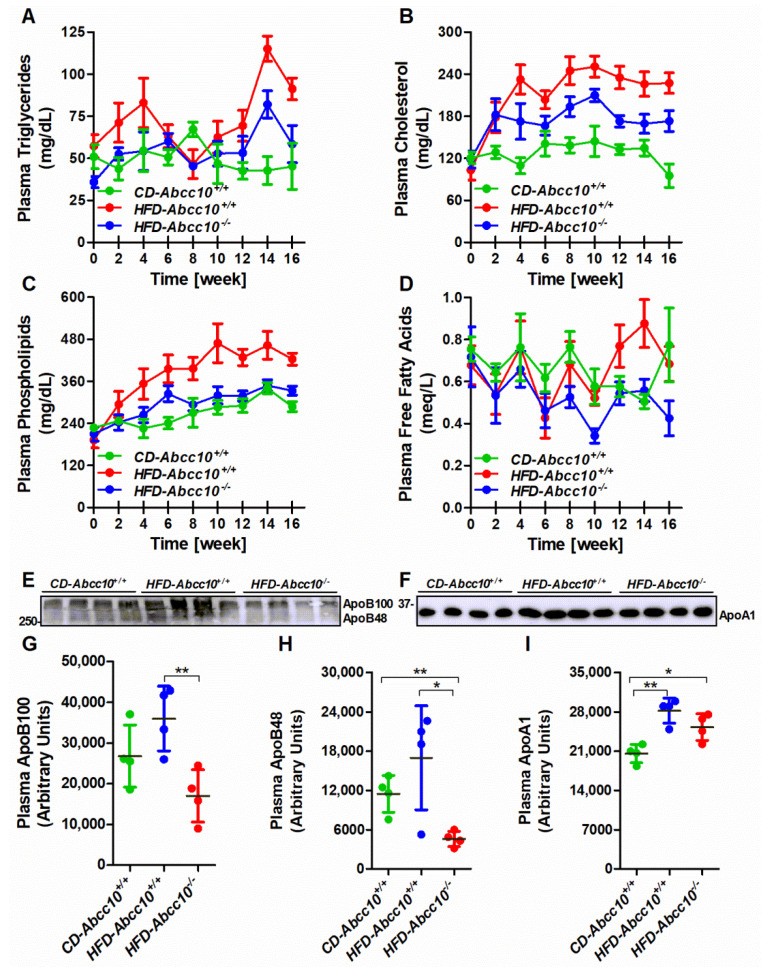
Deficiency of ABCC10 reduces plasma lipids and apoB lipoproteins in high-fat-diet-fed mice. Eight-week-old *Abcc10^+/+^* (*HFD-Abcc10^+/+^*, *n* = 4) and *Abcc10^−/−^* (*HFD-Abcc10^−/−^*, *n* = 5) male mice were fed high-fat obesity diet for 16 weeks. As a control, age-matched *Abcc10^+/+^* (*CD-Abcc10^+/+^*, *n* = 4) male mice were kept on chow diet during this period. Blood was collected every two weeks in the overnight-fasted mice and plasma was isolated to measure total triglycerides (**A**), cholesterol (**B**), phospholipids (**C**), and free fatty acids (**D**). Plasma at the termination of the experiment was also used to measure the levels of apoB (**E**) and apoA1 (**F**) lipoproteins by Western blotting. Density of the protein bands was quantified by using ImageJ software and the values were plotted (**G**–**I**). Values are plotted as replicates (mean ± SD). * *p* < 0.05 and ** *p* < 0.01.

We measured the bi-weekly changes in the levels of plasma lipids in *CD-Abcc10^+/+^* and *HFD-Abcc10^+/+^* and *HFD-Abcc10^−/−^* mice. As expected, we observed higher plasma triglyceride levels in *HFD-Abcc10^+/+^* mice compared to *CD-Abcc10^+/+^* mice (Figure 3A) for the initial few weeks, which was then reversed after 4 weeks of feeding. However, after 10 weeks of feeding, plasma triglycerides in *HFD-Abcc10^+/+^* mice again started to increase compared to *CD-Abcc10^+/+^* mice (Figure 3A). Similar to chow-diet-fed *Abcc10^−/−^* mice (Figure 1A), this increase in plasma triglycerides was lower in *HFD-Abcc10^−/−^* mice compared to *HFD-Abcc10^+/+^* mice (Figure 3A). There was also an increase in the levels of plasma cholesterol in *HFD-Abcc10^+/+^* mice compared to *CD-Abcc10^+/+^* mice, which became steady after 4 weeks of feeding (Figure 3B). This increase was lower in *HFD-Abcc10^−/−^* mice after 4 weeks of high-fat diet feeding compared to *HFD-Abcc10^+/+^* mice (Figure 3B). Similar to cholesterol, *HFD-Abcc10^+/+^* mice showed an increase in the levels of phospholipids during the feeding of the high-fat diet, which was prevented in ABCC10-deficient mice (Figure 3C). The levels of free fatty acids in the plasma were not different between the groups during the initial 8 weeks of feeding (Figure 3D). However, we observed lower levels of free fatty acids in *HFD-Abcc10^−/−^* mice compared to *HFD-Abcc10^+/+^* mice after 8 weeks of feeding (Figure 3D). The decrease in plasma lipid levels raised the obvious question of whether apoB lipoprotein secretion would be altered by the deletion of ABCC10 in mice. Analysis of apolipoproteins in the plasma at the end of the experiment showed that ApoB100 and ApoB48 levels were increased in *HFD-Abcc10^+/+^* mice compared to *CD-Abcc10^+/+^* (Figure 3E,G,H). Interestingly, we observed reduced levels of ApoB100 and ApoB48 in the plasma of *HFD-Abcc10^−/−^* mice (Figure 3E,G,H) compared to *HFD-Abcc10^+/+^*, which was consistent with the decreased lipid levels in the plasma of these mice. Again, plasma ApoA1 levels were increased in *HFD-Abcc10^+/+^* mice compared to *CD-Abcc10^+/+^* mice, but these levels remained unchanged in *HFD-Abcc10^−/−^* mice compared to *HFD-Abcc10^+/+^* mice (Figure 3F,I). These combined results suggest that ABCC10 deficiency leads to lower plasma lipid and apoB lipoprotein levels without changing apoA1 lipoprotein levels after high-fat-diet feeding in mice.

### 2.4. Plasma Glucose Levels Are Lower during Early Stages of High-Fat-Diet Feeding in ABCC10-Deficient Mice

**Figure 4:**
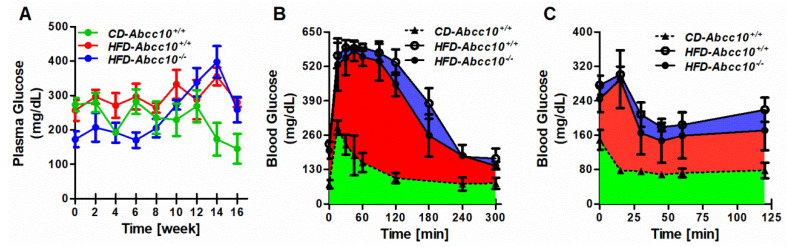
Deficiency of ABCC10 does not affect blood glucose, glucose tolerance, and insulin tolerance in high-fat-diet-fed mice. Eight-week-old *Abcc10^+/+^* (*HFD-Abcc10^+/+^*, *n* = 4) and *Abcc10^−/−^* (*HFD-Abcc10^−/−^*, *n* = 5) male mice were fed high-fat obesity diet for 16 weeks. As a control, age-matched *Abcc10^+/+^* (*CD-Abcc10^+/+^*, *n* = 4) male mice were kept on chow diet during this period. Blood was collected every two weeks in the overnight-fasted mice through tail vein bleeding, and plasma glucose was measured (**A**). After 14 weeks of feeding, GTT (**B**) and ITT (**C**) were performed in these mice and the area under the curve was plotted. Values are plotted as replicates (mean ± SD).

Prolonged high-fat-diet feeding is known to induce diabetes and insulin resistance in mice. To investigate whether ABCC10 deficiency has any effect on the development of diabetes during high-fat-diet feeding in mice, we measured the levels of plasma glucose every two weeks. Interestingly, *HFD-Abcc10^−/−^* mice showed lower plasma glucose levels compared to *CD-Abcc10^+/+^* or *HFD-Abcc10^+/+^* mice at the start of the feeding, which remained lower for up to 10 weeks of feeding (Figure 4A). However, after 10 weeks, we did not see any difference in the plasma glucose levels between *HFD-Abcc10^+/+^* and *HFD*-*Abcc10^−/−^* mice. Next, we performed glucose (GTT) and insulin (ITT) tolerance tests after 14 weeks of high-fat-diet feeding in these mice. Administration of glucose during GTT resulted in a significant increase in the blood levels in *HFD-Abcc10^+/+^* mice compared to *CD-Abcc10^+/+^* mice (Figure 4B) and a similar trend was observed in *HFD-Abcc10^−/−^* mice (Figure 4B). There was a longer delay in blood glucose to reach the basal levels in *HFD-Abcc10^+/+^* mice compared to *CD-Abcc10^+/+^* mice, and the area under the curve (AUC) was 121,058 ± 3810 (AUC, arbitrary units) and 34,905 ± 2672, respectively, between these two groups. *HFD-Abcc10^−/−^* mice showed a better response than *HFD-Abcc10^+/+^* mice to reach the basal levels, with an AUC of 106,268 ± 4332, which was significantly different to that of *HFD-Abcc10^+/+^* mice, *p =* 0.001 (Figure 4B). Similar observations were made when these mice were injected with insulin during the ITT measurements (Figure 4C). *HFD-Abcc10^−/−^* mice displayed a better response to insulin, with an AUC of 21,953 ± 2392, than *HFD-Abcc10^+/+^* mice, with an AUC of 25,860 ± 1315 (*p =* 0.022). Overall, these data indicate that the deletion of ABCC10 improves glucose tolerance and insulin sensitivity at the early stages in HFD-fed mice.

### 2.5. ABCC10 Deficiency Prevents Accumulation of Triglycerides in the Tissues

**Figure 5:**
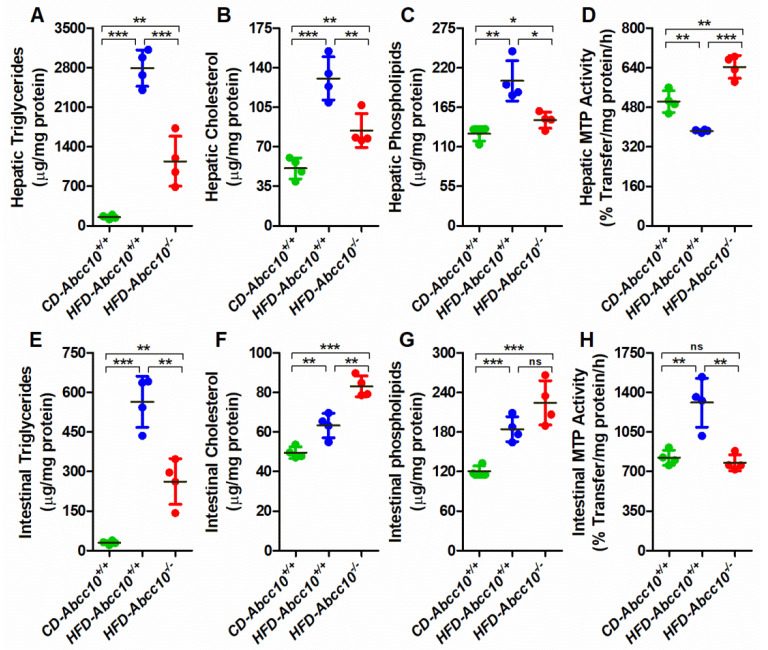
Deletion of *Abcc10* gene decreases plasma and tissue triglycerides in high-fat-diet-fed mice. Eight-week-old *Abcc10^+/+^* (*HFD-Abcc10^+/+^*, *n* = 4) and *Abcc10^−/−^* (*HFD-Abcc10^−/−^*, *n* = 5) male mice were fed high-fat obesity diet for 16 weeks. As a control, age-matched *Abcc10^+/+^ (CD-Abcc10^+/+^, n* = 4) male mice were kept on chow diet during this period. At the end of the experiment, mice were fasted overnight and sacrificed. Liver (**A**–**D**) and intestine (**E**–**H**) were collected and used to measure triglycerides (**A**,**E**), cholesterol (**B**,**F**), phospholipids (**C**,**G**), and MTP activity (**D**,**H**). Values are plotted as replicates (mean ± SD). * p < 0.05, ** *p* < 0.01, and *** *p* < 0.001.

**Figure 6:**
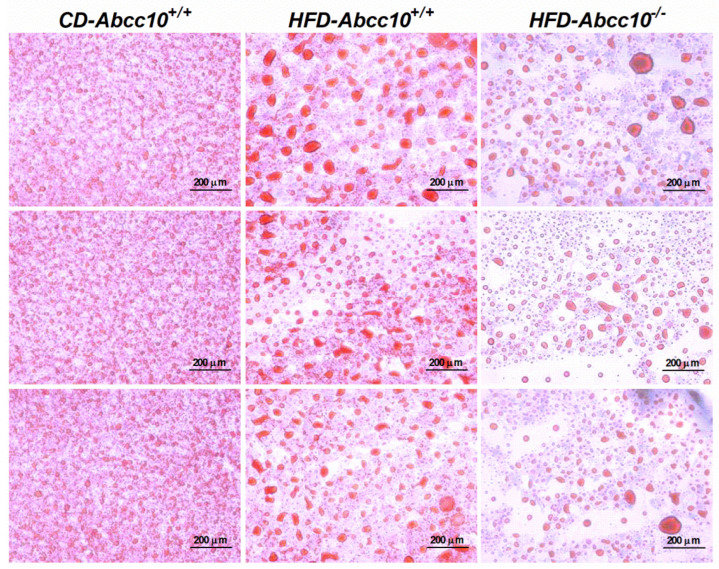
Deletion of *Abcc10* gene reduces accumulation of lipids in high-fat-diet-fed mice. Eight-week-old *Abcc10^+/+^* (*HFD-Abcc10^+/+^*, *n* = 4) and *Abcc10^−/−^* (*HFD-Abcc10^−/−^*, *n* = 5) male mice were fed high-fat obesity diet for 16 weeks. As a control, age-matched *Abcc10^+/+^* (*CD-Abcc10^+/+^*, *n* = 4) male mice were kept on chow diet during this period. At the end of the experiment, mice were fasted overnight and sacrificed. Oil Red O staining for lipid droplets in liver tissue sections from these mice was performed and representative pictographs (10×) from each group are shown in the figure.

As stated earlier, the liver and intestine play an important role in maintaining the homeostasis of plasma lipids. Therefore, we measured the changes in the lipid levels in the liver and intestine of these mice. As expected, feeding of the high-fat diet led to a 17-fold increase in the accumulation of triglycerides in the livers of *HFD-Abcc10^+/+^* mice compared to *CD-Abcc10^+/+^* mice (Figure 5A). This increase in the accumulation of liver triglycerides was prevented by 59% in *HFD-Abcc10^−/−^* mice (Figure 5A), suggesting that the absence of ABCC10 plays a protective role in the development of liver steatosis. Oil Red O staining also demonstrated that deletion of the *Abcc10* gene contributed to the lower content of lipid droplets in the livers of *HFD-Abcc10^−/−^* mice (Figure 6), suggesting that these mice accumulate less neutral lipids such as triglycerides and cholesterol esters in the liver. Similar to triglycerides, we saw a significant increase of 155% in the levels of hepatic cholesterol in *HFD-Abcc10^+/+^* mice compared to *CD-Abcc10^+/+^* mice (Figure 5B). This increase was reduced by 35% in *HFD-Abcc10^−/−^* mice compared to *HFD-Abcc10^+/+^* mice. Hepatic phospholipids were also increased by 57% in *HFD-Abcc10^+/+^* mice compared to *HFD-Abcc10^+/+^* mice, which were again decreased by 27% in *HFD-Abcc10^−/−^* mice compared to *HFD-Abcc10^+/+^* mice (Figure 5C). Since microsomal triglyceride transfer protein (MTP) plays an important role in the transport of lipids, we also determined its activity in the livers of these mice. Our data showed that feeding of a high-fat diet results in a significant decrease in the hepatic MTP activity levels in *HFD-Abcc10^+/+^* mice (Figure 5D). On the other hand, we saw a significant increase in the activity of hepatic MTP in *HFD-Abcc10^−/−^* mice (Figure 5D). These combined data suggest that deletion of ABCC10 prevents the accumulation of lipids, especially triglycerides, in the liver and, at the same time, increases the activity of hepatic MTP.

Next, we determined the levels of lipids in the intestinal tissues. Similar to hepatic lipids, there was a significant increase in the levels of intestinal triglycerides (Figure 5E), cholesterol (Figure 5F), and phospholipids (Figure 5G) in *HFD-Abcc10^+/+^* mice compared to *CD-Abcc10^+/+^* mice. Consistent with hepatic triglycerides, deficiency of ABCC10 prevented the accumulation of intestinal triglycerides by 54% in *HFD-Abcc10^−/−^* mice compared to *HFD-Abcc10^+/+^* mice (Figure 5E). Contrary to triglycerides, we observed a further increase of 31% in cholesterol (Figure 5F) and 22% in phospholipids (Figure 5G) in *HFD-Abcc10^−/−^* mice compared to *HFD-Abcc10^+/+^* mice. Determination of MTP activity showed a significant increase in the intestines of *HFD-Abcc10^+/+^* mice compared to *CD-Abcc10^+/+^* mice, which was reduced in *HFD-Abcc10^−/−^* mice (Figure 5H). These combined data suggest that deletion of ABCC10 decreases intestinal MTP activity, leading to the accumulation of cholesterol and phospholipids. Furthermore, decreased accumulation of triglycerides in the intestine may be due to decreased uptake by the enterocytes.

### 2.6. Deletion of ABCC10 in Mice Alters Expression of Several Lipid Metabolism Genes in the Liver and the Intestine

**Figure 7:**
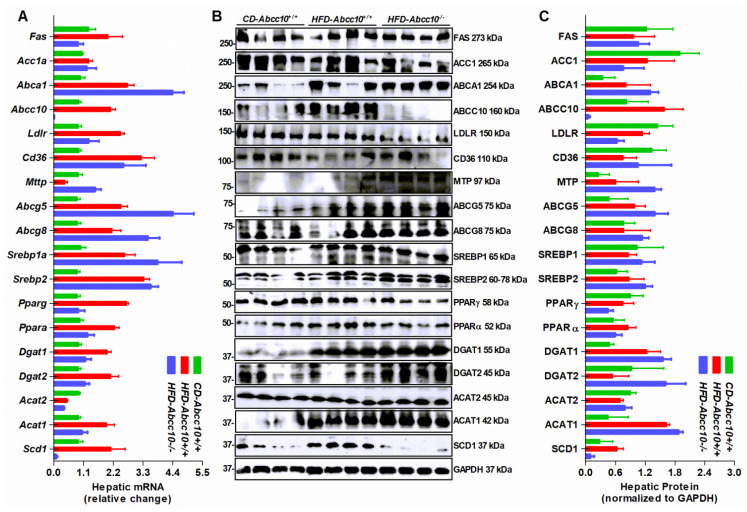
Expression of lipid metabolism genes in the liver is altered in *Abcc10*-gene-deleted high-fat-diet-fed mice. Eight-week-old *Abcc10^+/+^* (*HFD-Abcc10^+/+^*, *n* = 4) and *Abcc10^−/−^* (*HFD-Abcc10^−/−^*, *n* = 5) male mice were fed high-fat obesity diet for 16 weeks. As a control, age-matched *Abcc10^+/+^* (*CD-Abcc10^+/+^*, *n* = 4) male mice were kept on chow diet during this period. At the end of the experiment, mice were fasted overnight and sacrificed. Livers were used to isolate total RNA to measure the expression of genes using quantitative real-time PCR (**A**). Livers were also homogenized and used to measure the expression of proteins by Western blotting (**B**). Density of the protein bands was quantified by using ImageJ software and the values were plotted after normalizing to the internal GAPDH control (**C**). Values are plotted as replicates (mean ± SD).

Since we observed that ABCC10 deletion affects lipid metabolism in the plasma and tissues, we assessed its effect on the expression of various lipid metabolism genes in the liver and the intestine. Our data suggested that feeding of a high-fat diet to mice leads to an increase in the hepatic expression of cholesterol efflux genes such as *Abca1*, *Abcg5*, and *Abcg8* in *HFD-Abcc10^+/+^* mice compared to *CD-Abcc10^+/+^* mice, which was further increased in *HFD-Abcc10^−/−^* mice (Figure 7A–C). A similar trend was observed in *Acat1* (Figure 7A–C) but there was a decrease in the expression of *Acat2* mRNA levels in *HFD-Abcc10^+/+^* and *HFD-Abcc10^−/−^* mice compared to *CD-Abcc10^+/+^* mice (Figure 7A). However, this decrease was not reflected in the ACAT2 protein levels (Figure 7B,C). We also observed an increase in the expression of hepatic mRNA and the protein levels of the *Abcc10* gene in *HFD-Abcc10^+/+^* mice compared to *CD-Abcc10^+/+^* mice (Figure 7A–C). The expression of most of the triglyceride and fatty acid metabolism genes, such as *Fas*, *Ldlr*, *Cd36*, *Pparg*, *Ppara*, *Dgat1*, *Dgat2*, and *Scd1*, showed an increase in the mRNA expression levels in *HFD-Abcc10^+/+^* mice compared to *CD-Abcc10^+/+^* mice, and this increase was prevented in *HFD-Abcc10^−/−^* mice (Figure 7A). The protein levels of these genes did not correlate with the mRNA levels, except for SCD1, which was increased in *HFD-Abcc10^+/+^* mice and decreased in *HFD-Abcc10^−/−^* mice (Figure 7B,C). These data suggest that deletion of ABCC10 in mice alters the expression of several lipid metabolism genes in the liver.

**Figure 8:**
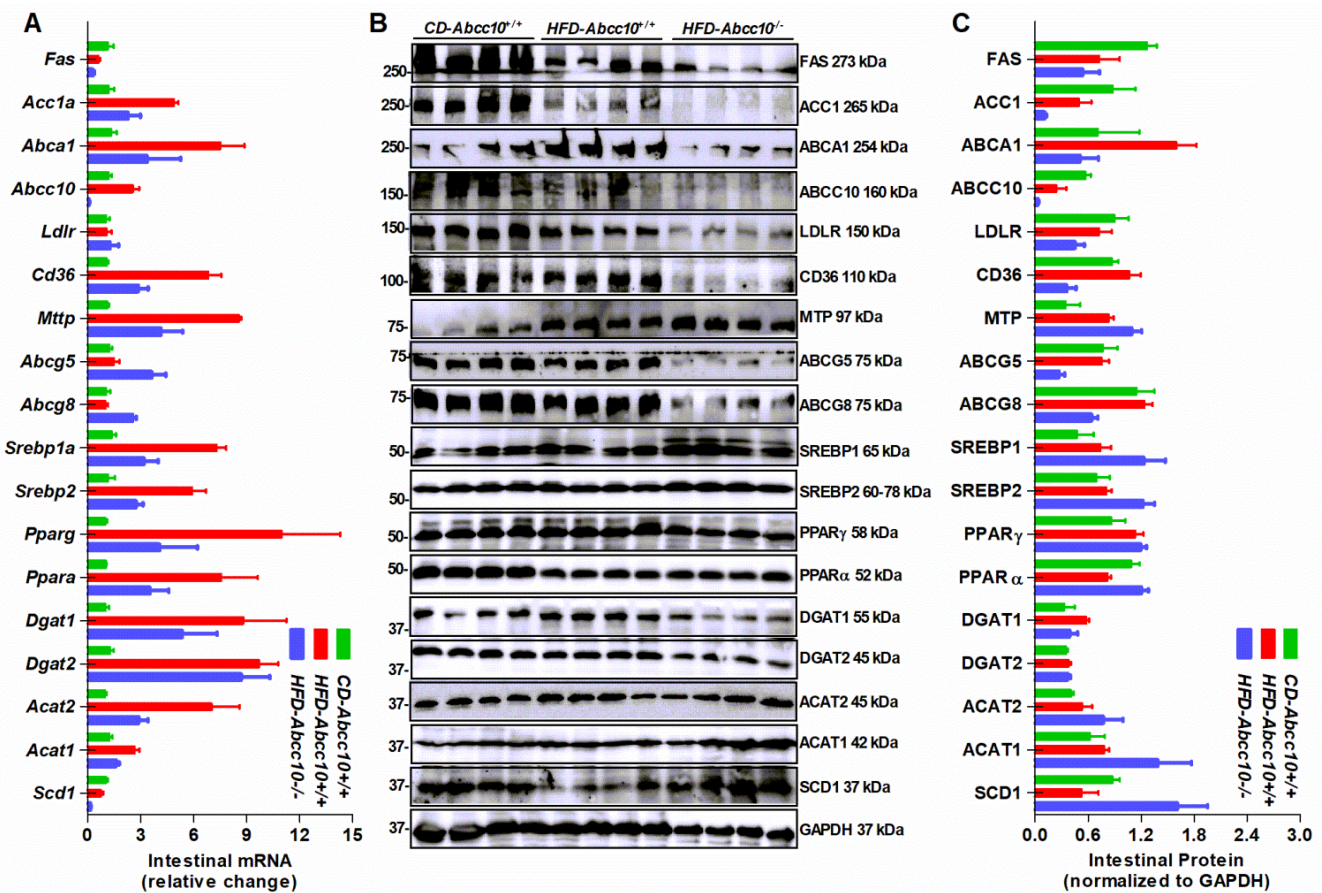
Expression of lipid metabolism genes in the intestine is altered in *Abcc10*-gene-deleted high-fat-diet-fed mice. Eight-week-old *Abcc10^+/+^* (*HFD-Abcc10^+/+^*, *n* = 4) and *Abcc10^−/−^* (*HFD-Abcc10^−/−^*, *n* = 5) male mice were fed high-fat obesity diet for 16 weeks. As a control, age-matched *Abcc10^+/+^* (*CD-Abcc10^+/+^*, *n* = 4) male mice were kept on chow diet during this period. At the end of the experiment, mice were fasted overnight and sacrificed. Intestines were used to isolate total RNA to measure the expression of genes using quantitative real-time PCR (**A**). Intestines were also homogenized and used to measure the expression of proteins by Western blotting (**B**). Density of the protein bands was quantified by using ImageJ software and the values were plotted after normalizing to the internal GAPDH control (**C**). Values are plotted as replicates (mean ± SD).

Next, we measured the changes in the expression of lipid metabolism genes in the intestine (Figure 8). Feeding of a high-fat diet to mice increased the expression of cholesterol efflux gene *Abca1*, without changing the expression of *Abcg5* and *Abcg8* in *HFD*-*Abcc10^+/+^* mice compared to *CD-Abcc10^+/+^* mice (Figure 8A–C). This increase in Abca1 mRNA and protein expression was prevented in *HFD-Abcc10^−/−^* mice (Figure 8A–C). Similarly, mRNA expression of *Acat1* and *Acat2* in the *HFD-Abcc10^+/+^* mice intestine was increased, and this increase was prevented in *HFD-Abcc10^−/−^* mice (Figure 8A). We did not see much difference in the protein levels of ACAT1 and ACAT2 in these mice (Figure 8B,C). Again, there was an increase in the expression of intestinal mRNA of *Abcc10* in *HFD-Abcc10^+/+^* mice compared to *CD-Abcc10^+/+^* mice (Figure 8A) but its protein levels were decreased (Figure 8B,C). The expression of triglyceride and fatty acid metabolism genes such as *Acc1a*, *Cd36*, *Srebp1a*, *Pparg*, *Ppara*, *Dgat1*, and *Dgat2* showed an increase in *HFD-Abcc10^+/+^* mice compared to *CD-Abcc10^+/+^* mice, and, except for *Dgat2*, this increase was prevented in *HFD-Abcc10^−/−^* mice (Figure 8A). The protein levels of most of these genes did not change in *HFD-Abcc10^+/+^* mice compared to *CD-Abcc10^+/+^* mice, but we saw a decrease in FAS, ACC1, and CD36 levels in *HFD-Abcc10^−/−^* mice compared to *HFD-Abcc10^+/+^* mice (Figure 8B,C). There was a decrease in the protein levels of SCD1 in *HFD-Abcc10^+/+^* mice and an increase in *HFD-Abcc10^−/−^* mice compared to *CD-Abcc10^+/+^* mice (Figure 8B,C). These data suggest that deletion of ABCC10 in mice alters the expression of several lipid metabolism genes in the intestine. Overall, *HFD-Abcc10^−/−^* mice displayed a significant reduction in the expression of lipid metabolism genes compared to *HFD-Abcc10^+/+^* mice.

### 2.7. Deletion of ABCC10 in Mice Affects Absorption of Triglycerides in the Intestine

**Figure 9:**
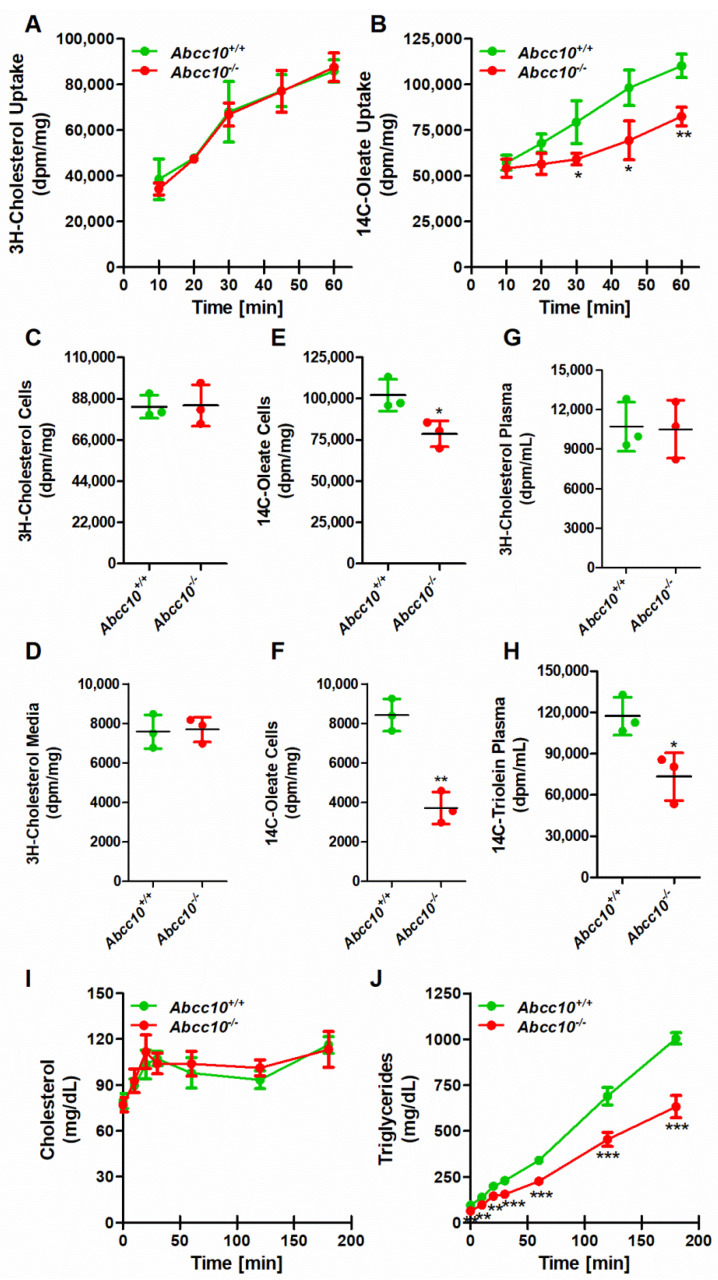
Absorption and secretion of triglycerides is decreased in *Abcc10*-gene-deleted mice. Chow-diet-fed, 12-week-old *Abcc10^+/+^* and *Abcc10^−/−^* male mice (*n* = 3) were fasted overnight and used to isolate enterocytes. Enterocytes were labeled in DMEM media containing 0.5 μCi/mL of 3H-cholesterol or 14C-oleic acid and incubated at 37 °C. Cells were collected every 10 min and washed. Lipids were extracted to study the uptake of 3H-cholesterol (**A**) or 14C-oleic acid (**B**). After 1 h, enterocytes were washed and lipids were isolated to determine uptake of radiolabeled lipids. To study secretion of lipoproteins, enterocytes were isolated from overnight-fasted mice (*n* = 3) and labeled for 1 h with 0.5 μCi/mL of 3H-cholesterol (**C**,**D**) or 14C-oleic acid (**E**,**F**). Enterocytes were washed and incubated with fresh media containing 1.4 mM oleic acid containing micelles. After 2 h, enterocytes were centrifuged and supernatants were collected to measure radiolabeled lipids in the media (**D**,**F**). Pellets were washed and lipids were isolated to determine the remaining cellular radiolabeled lipids (**C**,**E**). Age-matched 12-week-old chow-diet-fed *Abcc10^+/+^* and *Abcc10^−/−^* male mice (*n* = 3) were fasted overnight and injected intraperitoneally with P407 (30 mg/mouse). After 1 h, mice were fed 0.5 μCi of 3H-cholesterol (**G**) or 14C-triolein (**H**) as well as 0.2 mg of cholesterol in 15 μL of olive oil. Plasma was collected after 2 h and radiolabeled lipids were measured. *Abcc10^+/+^* and *Abcc10^−/−^* male mice (*n* = 4) fed a chow diet for 12 months were fasted overnight and injected intraperitoneally with P407 (30 mg/mouse). Blood was withdrawn at the indicated time points for 3 h. Cholesterol (**I**) and triglycerides (**J**) were measured at each time and plotted against time. Values are plotted as replicates (mean ± SD). * p < 0.05, ** *p* < 0.01, and *** *p* < 0.001, as compared with *Abcc10^+/+^* mice.

We have observed that the deletion of ABCC10 affects plasma and tissue triglycerides in mice. Since we observed lower triglycerides in the intestines, we hypothesized that the decrease in the levels of intestinal triglycerides in *Abcc10^−/−^* mice could be due to the lower absorption of lipids by the intestine. To test this possibility, we isolated enterocytes from *Abcc10^+/+^* and *Abcc10^−/−^* mice and studied the uptake and secretion of radiolabeled cholesterol and oleate. Incubation of enterocytes with 3H-cholesterol for up to 60 min did not show any significant difference in the uptake between *Abcc10^+/+^* and *Abcc10^−/−^* mice (Figure 9A). In contrast, there was a significant reduction in the uptake of 14C-oleate after 20 min of incubation in *Abcc10^−/−^* mice compared to *Abcc10^+/+^* mice (Figure 9B), suggesting that intestinal cells may absorb fewer triglycerides in the absence of ABCC10. Our data showed that ABCC10-deficient mice have lower plasma triglycerides, which may be due to decreased secretion of lipids into the circulation from the intestines. To test this, we pulsed the enterocytes isolated from *Abcc10^+/+^* and *Abcc10^−/−^* mice with 3H-cholesterol and 14C-oleate for 1 h and, after washing, cells were chased with fresh DMEM media containing 1.4 mM oleic acid for 2 h. We did not see any significant change in the cellular (Figure 9C) or secreted (Figure 9D) cholesterol levels between *Abcc10^+/+^* and *Abcc10^−/−^* mice, suggesting that ABCC10 deficiency does not affect cholesterol uptake or secretion. However, there was a significant reduction of 23% and 56% in the levels of cellular (Figure 9E) and secreted (Figure 9F) oleate, respectively, in *Abcc10^−/−^* mice compared to *Abcc10^+/+^* mice. These data suggest that deletion of ABCC10 only affects the uptake and secretion of triglycerides in the intestinal cells and does not play any major role in cholesterol uptake or secretion.

To provide direct evidence of whether the deletion of ABCC10 affects the absorption of triglycerides in the intestine, *Abcc10^+/+^* and *Abcc10^−/−^* mice were fasted for 16 h and intraperitoneally injected with P407, a potent lipoprotein lipase, to inhibit apoB lipoprotein clearance in the plasma. After 1 h, mice were gavaged with 3H-cholesterol and 14C-triolein in corn oil and their appearance in the plasma was measured after 2 h of gavage. As seen before, there was no significant difference in the plasma levels of 3H-cholesterol between *Abcc10^+/+^* and *Abcc10^−/−^* mice (Figure 9G). However, *Abcc10^−/−^* mice showed a significant reduction of 38% in the levels of 14C-triolein in the plasma compared to *Abcc10^−/−^* mice after 2 h of gavage (Figure 9H). These data suggest that deletion of ABCC10 is involved in the absorption of triglycerides in mice. To determine if the liver also contributes to decreased plasma triglyceride levels, we fasted *Abcc10^+/+^* and *Abcc10^−/−^* mice for 16 h and then injected them intraperitoneally with P407. Levels of cholesterol and triglycerides secreted by the liver into the plasma were measured at different time periods. Since we did not provide any exogenous source of lipids to these mice during these experiments, any change in plasma lipid levels in these mice will be due to the contribution from the liver. There was no significant difference in the levels of cholesterol in the plasma of *Abcc10^+/+^* and *Abcc10^−/−^* mice (Figure 9I). On the other hand, the secretion of triglycerides by the liver was significantly reduced in the plasma of *Abcc10^−/−^* mice compared to *Abcc10^+/+^* mice (Figure 9J). This decrease in secretion may be explained by the reduction in lipid synthesis in the liver resulting from the lower expression of lipid-synthesizing genes in *Abcc10^−/−^* mice (Figure 7). It is also likely that lower hepatic VLDL secretion in *Abcc10^−/−^* mice may contribute to lower plasma triglyceride levels.

### 2.8. ABCC10 Deficiency Does Not Affect Atherosclerosis in Western-Type-Diet-Fed ApoE Knockout Mice

**Figure 10:**
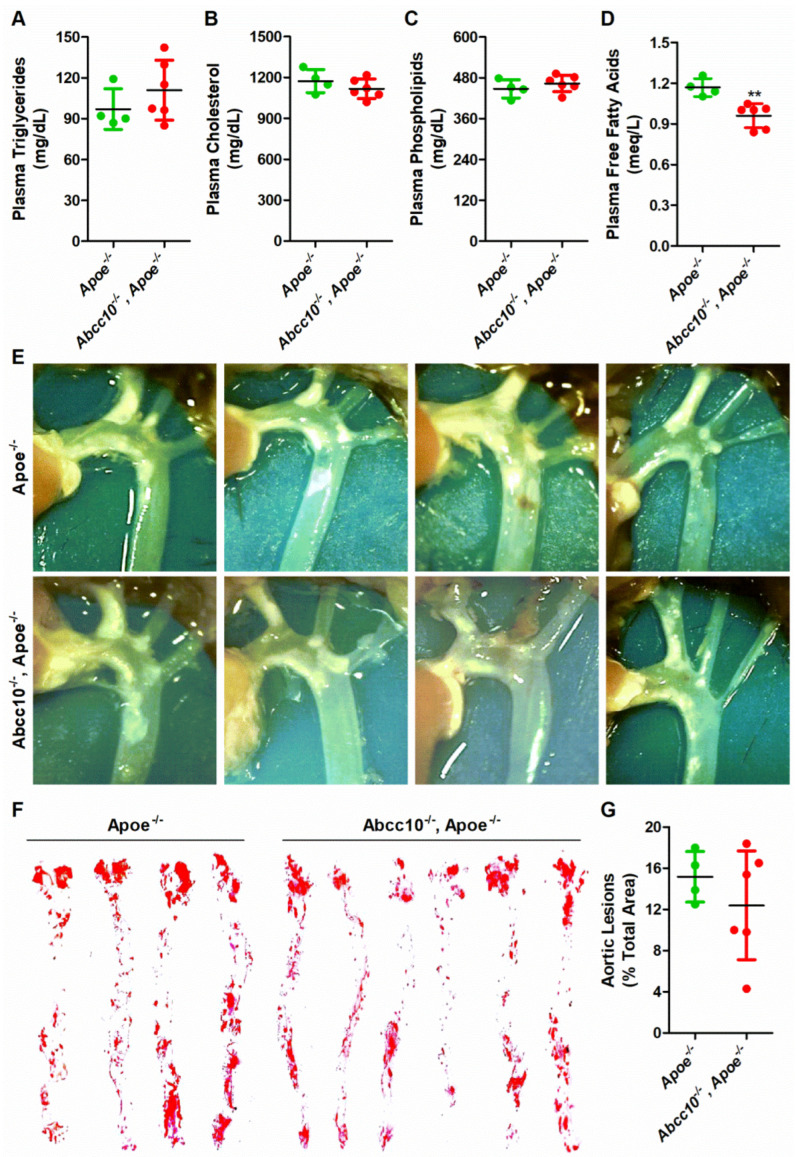
Ablation of ABCC10 in *Apoe^−/−^* mice does not enhance atherosclerosis. Age-matched *Apoe^−/−^* mice (*n* = 4) and *Abcc10^−/−^*, *Apoe^−/−^* (*n* = 6) were fed Western-type diet for 12 weeks. At the end of the experiment, mice were fasted overnight and blood was collected to isolate plasma. Total triglycerides (**A**), cholesterol (**B**), phospholipids (**C**), and free fatty acids (**D**) were measured. Aortic arch and other proximal arteries were dissected and photographed. Representative photographs from each group are shown (**E**). Aortas were isolated, stained with Oil Red O (**F**), and quantified (**G**). Values are plotted as replicates (mean ± SD). ** *p* < 0.01 as compared with *Apoe^−/−^* mice.

Our data indicated that ABCC10 deficiency affects plasma and tissue lipid levels. Since changes in lipid levels are associated with the development of atherosclerosis, and bioinformatics analysis of genes expressed in samples of atherosclerotic lesions has been shown to increase the expression of ABCC10, we wanted to see if ABCC10 deficiency affects atherosclerosis. To study this, we crossed *Abcc10^−/−^* mice with *Apoe^−/−^* mice to create double-knockout (*Abcc10^−/−^, Apoe^−/−^*) mice. Aged-matched *Apoe^−/−^* and double-knockout *Abcc10^−/−^, Apoe^−/−^* mice were fed a Western-type diet for 12 weeks and plasma lipids were measured. Interestingly, we did not see any significant difference in the plasma triglycerides (Figure 10A), cholesterol (Figure 10B), and phospholipids (Figure 10C) between *Abcc10^−/−^, Apoe^−/−^* double-knockout, and *Apoe^−/−^* mice. However, there was a significant decrease of around 18% in the levels of free fatty acids in the plasma of *Abcc10^−/−^* and *Apoe^−/−^* double-knockout compared to *Apoe^−/−^* mice (Figure 10D). Furthermore, we did not observe any major difference in the extent of atherosclerotic plaques between the two groups (Figure 10E). *En face* analyses of Oil-Red-O-stained thoracic aortic areas also did not exhibit any significant change in the accumulation of lipids in the atherosclerotic lesions of *Abcc10^−/−^* and *Apoe^−/−^* double-knockout mice compared to *Apoe^−/−^* mice (Figure 10F–G). These studies indicate that ABCC10 deficiency did not reduce the atherosclerotic plaques in Western-type-diet-fed mice.

## 3. Discussion

The intestine and liver play an essential role in regulating plasma lipid and lipoprotein levels. Our study shows that deletion of the *Abcc10* gene in mice reduces the accumulation of triglycerides in the intestine and the liver (Figure 1). Our data also indicate that ABCC10 may be involved in regulating the levels of other lipids in the plasma and tissues. Feeding of a high-fat diet to wildtype mice (*HFD-Abcc10^+/+^*) resulted in an increase in body weight gain and was consistent with other published reports [28,29,30]. In the current study, ablation of ABCC10 prevented body weight gain in HFD-fed mice. Our data indicate that deletion of ABCC10 decreases plasma and tissue triglycerides in chow-diet- and high-fat-diet-fed mice. However, ABCC10 deletion did not alter the levels of cholesterol and phospholipids in the intestines of these mice. To our knowledge, this is the first report that suggests that ABBC10 regulates plasma and tissue lipid levels in mice.

Enterocytes and radiolabeled studies suggest that one of the reasons for the reduction in plasma triglycerides may be due to defective fatty acid uptake and absorption. Our lipoprotein lipase inhibitor experiments suggest that the liver may also contribute to lower plasma triglyceride levels. In contrast, the current study showed that ABCC10 deficiency has no effect on cholesterol uptake or secretion by the enterocytes isolated from chow-fed mice. We have previously shown that one of the molecular mechanisms behind increased intestinal lipid absorption was enhanced MTP expression [31]. In the current study, lower expression and activity of MTP in the intestines of *Abcc10^−/−^* mice may have contributed to the lower plasma apoB48 lipoproteins and triglycerides. The reduced intestinal MTP expression and activity may also be a secondary effect of the lower uptake and synthesis of fatty acids by enterocytes. It is likely that lower hepatic apoB-containing VLDL lipoprotein secretion in *Abcc10^−/−^* mice may also contribute to lower plasma triglyceride levels. This is supported by our finding in the liver that shows a significant decrease in plasma triglyceride secretion in *Abcc10^−/−^* mice after inhibiting lipoprotein clearance by P407. Increased expression and activity of MTP in the liver may be a compensatory mechanism to enhance lipoprotein secretion rates but, due to lower hepatic lipid synthesis, sufficient lipids may not be secreted in the circulation. In intestine-specific MTP knockout mice, which had lower plasma lipid levels, it was shown that the secretion of hepatic triglycerides via ApoB100-containing VLDL particles was increased due to compensatory alterations in the distinct organ of gene manipulation [32]. Chylomicron secretion from the intestine has been shown to considerably decline in apobec-1-knockout mice, suggesting that ApoB48 exerts its role in the early stage of chylomicron assembly [33]. Our data indicate that deletion of ABCC10 results in lower ApoB100 and ApoB48 lipoprotein levels in the plasma, suggesting that both the liver and intestine may contribute to lower triglyceride levels.

Although fasting plasma glucose in *HFD-Abcc10^−/−^* mice was not considerably different from that in *HFD-Abcc10^+/+^* mice, these mice showed a better response to insulin and glucose. A decrease in lipid absorption in *Mgat2* knockout mice has been shown to be protective against high-fat-diet-induced obesity and insulin resistance [34,35]. Similar observations were made in *Park2* gene knockout mice [36], which support our data suggesting that impaired intestinal lipid absorption in ABCC10 knockout mice may reduce the metabolic load to peripheral organs and, therefore, protects the mice from high-fat-diet-induced insulin resistance. *Abcc10* gene knockout mice appear normal on a regular chow diet but are protected from high-fat-diet-induced obesity similarly to *Mgat2* and *Park2* knockout mice [34,35,36], and their plasma triglyceride levels are also decreased [37,38]. This decreased plasma triglyceride phenotype seems to result from the reduced absorption of lipids [35,37,38]. There is a possibility that these mice may consume less food or have increased energy expenditure or activity (data not determined) [38,39]. We can speculate that, since we observed an increase in the mass of brown fat in *HFD-Abcc10^−/−^* mice, they may have increased energy expenditure. However, this needs to be studied further.

Determination of mRNA and protein expression in the liver and intestine of high-fat-diet-fed mice suggested that ABCC10 ablation affects lipid metabolism genes. Interestingly, the expression of cholesterol efflux genes such as *Abca1*, *Abcg5*, and *Abcg8* was significantly upregulated in the livers of high-fat-diet-fed *Abcc10^−/−^* mice, suggesting that the efflux of cholesterol by hepatocytes may be increased in these mice. This may also explain the lower hepatic cholesterol levels found in *HFD-Abcc10^−/−^* mice (Figure 5B). On the other hand, the expression of fatty acid and triglyceride metabolism genes was lower in *HFD-Abcc10^−/−^* mice, which may explain the lower triglyceride content in the liver (Figure 5A). A reduction in the expression of *Abca1* mRNA and protein in *HFD-Abcc10^−/−^* mice may be responsible for the increased accumulation of cholesterol and phospholipids in the intestine. As expected, mRNA expression of triglyceride and fatty acid metabolism genes in the intestine increased after high-fat-diet feeding in *HFD-Abcc10^+/+^* mice. Interestingly, this increase was attenuated in *HFD-Abcc10^−/−^* mice, suggesting that these mice are synthesizing less triglycerides in the intestine. Our data clearly suggest that *Abcc10^−/−^* mice absorb and synthesize less triglycerides in the intestine, leading to lower plasma triglycerides. It is possible that the reduction in hepatic triglycerides may be due to decreased uptake from the plasma because of reduced absorption in the intestine. We have shown previously that the reduced absorption of lipids in the intestine of chow-fed *I-Ire1a^−/−^* mice leads to compensatory changes in the liver to boost the synthesis due to less lipids coming from the intestine [40]. Similar observations have been made in intestine-specific MTP knockout mice, which show a decrease in hepatic lipid levels with a compensatory increase in lipogenic genes due to less lipid absorption from the intestine [32,41]. In the current study, changes in the expression of several lipid metabolism genes in the liver of *HFD-Abcc10^−/−^* mice may not be secondary to the changes in lipid absorption by the intestine, since we did not see any compensatory increase in lipid-synthesizing genes (Figure 7), as was observed in other studies [32,40,41].

Atherosclerosis is a multifactorial disease and hyperlipidemia is one of the known risk factors for cardiovascular disease [42]. A bioinformatics analysis of genes expressed in samples of atherosclerotic lesions and control arteries without atherosclerotic lesions showed increased expression of ABCC10 [27], suggesting a role of ABCC10 in atherogenesis. Therefore, we hypothesized that lower hyperlipidemia and the absence of ABCC10 in *Abcc10^−/−^* mice might reduce atherosclerosis in *Apoe^−/−^* mice. Contrary to the high-fat diet, a Western-type diet did not cause any significant change in the plasma lipids of *Abcc10^−/−^, Apoe^−/−^* double-knockout mice, except for reduced free fatty acid levels. Interestingly, *Abcc10* gene deletion did not alter triglyceride levels in ApoE-deficient mice, indicating that ABCC10-mediated effects on triglyceride metabolism require ApoE. We also did not see any significant difference in the extent of plaques or *en face* staining with Oil Red O in thoracic aortic areas between *Apoe^−/−^* and *Abcc10^−/−^*, *Apoe^−/−^* double-knockout mice, suggesting that ABCC10 deficiency does not affect atherosclerosis. The observation of increased gene expression of ABCC10 in samples of atherosclerotic lesions [27] may be a secondary effect and not reflect any change in the ABCC10 protein levels. Therefore, changes in ABCC10 levels in our study may not have had any direct biochemical effect on the development of atherosclerosis in mice. The finding that plasma triglycerides were not changed in ABCC10-deficient mice on an ApoE background was very intriguing and needs further investigation.

In summary, these studies show that ABCC10 deficiency ameliorates diet-induced obesity in mice. The reduction in body weight and plasma lipid levels may be due to reduced absorption of triglycerides by the intestine. It is likely that agents that inhibit the activity of ABCC10 might reduce hyperlipidemia and obesity.

## 4. Materials and Methods

### 4.1. Materials

Infinity Cholesterol (catalog #TR13421), Infinity Triglyceride (catalog #TR22421), and TRIzol^TM^ (catalog #15596018) reagents were purchased from Thermo Fisher Scientific (Middletown, VA, USA). Autokit Glucose (catalog #997-03001), Phospholipids C (catalog #997-01801), and HR Series NEFA-HR(2) (catalog #999-34691, 995-34791, 991-34891, and 993-35191) kits were purchased from Fujifilm Wako Chemicals USA (Richmond, VA, USA). Omniscript RT (catalog #205113) kit was purchased from Qiagen (Germantown, MD, USA) and qPCR^TM^ core kit for SYBR Green I (catalog #10-SN10-05) was from Eurogentec (San Diego, CA, USA). ^3^H-cholesterol (catalog #NET139001MC), ^14^C-oleic acid (catalog #NEC317250UC), and ^14^C-triolein (catalog #NEC674250UC) were from PerkinElmer (Shelton, CT, USA). Poloxamer 407 (catalog #P1166) was purchased from Spectrum Chemical (New Brunswick, NJ, USA). Primary and secondary antibodies were purchased from either Cell Signaling (Danvers, MA, USA) or Abcam (Cambridge, MA, USA). All other chemicals and solvents were obtained from Fisher Scientific through its local distributor in the Kingdom of Saudi Arabia.

### 4.2. Animals and Diets

ABCC10-deficient (*Abcc10^−/−^*) mice on a C57Bl/6J background [43] were transferred from the laboratory of Dr. M. Mahmood Hussain, NYU Langone Health, Long Island through a material transfer agreement. These mice were originally received as a kind gift from Dr. Elizabeth Hopper-Borge, Fox Chase Cancer Center, Philadelphia. ApoE-deficient (*Apoe^−/−^*) mice on a C57Bl/6J background were transferred from Dr. Xiaoyue Pan, NYU Langone Health, Long Island through a material transfer agreement, which were originally purchased from Jackson Laboratory (Bar Harbor, ME, USA). For the atherosclerosis study, *Abcc10^−/−^* mice were crossed with *Apoe^−/−^* mice to create double-knockout (*Abcc10^−/−^*, *Apoe^−/−^*) mice. Mice were housed in a temperature-controlled, specific pathogen-free room at 22 ± 0.5 °C with a 12-h lighting schedule (700–1900 h). Mice were kept on a chow diet (10% energy by fat, catalog #D12450B, Research Diets, Inc., New Brunswick, NJ, USA) or fed a high-fat diet (60% energy by fat, catalog #D12492, Research Diets, Inc., New Brunswick, NJ, USA) for 16 weeks or a Western-type diet (0.15% cholesterol, 20% saturated fat, catalog #D12079B, Research Diets, Inc., New Brunswick, NJ, USA) for 12 weeks. Only male mice were used in these experiments to avoid the effects of hormonal changes on plasma lipids in female mice. Experiments were conducted with the approval of KAIMRC Institutional Animal Care and Use Committee (Protocol #s RA17-013-A and RA20-005-A).

### 4.3. Lipid Measurements and Oil Red O Staining

Total cholesterol, triglycerides, phospholipids, and free fatty acid levels in the plasma and tissues were measured using commercially available kits, as described previously [44]. For histology [41], liver tissues were fixed overnight in 10% formalin, dehydrated in 30% sucrose, embedded in M1 cryo-preservation media at −20 °C, and stored at −70 °C. Sections (7 µm) were placed on Tissue-Tack (Polysciences Inc. Warrington, PA, USA) slides, dehydrated in 60% isopropyl alcohol, and immersed in 1% Oil Red O (catalog #154–02072) from Wako (Richmond, VA, USA) for 30 min at 22 °C. Slides were washed in 60% isopropyl alcohol, rinsed with tap water for 10 s, counterstained with Gill’s hematoxylin for at least 20 min, rinsed with tap water until clear, acidified in alcohol (0.4% HCl in 95% ETOH), rinsed with tap water again, and dipped in basic solution (0.03 N NaOH). Images were taken with the Brightfield EVOS FL Cell Imaging System using 10× magnifications (ThermoFisher Scientific, Waltham, MA, USA).

### 4.4. Glucose Analysis, Glucose and Insulin Tolerance Tests

Whole-body plasma glucose levels were measured in *Abcc10^+/+^* and *Abcc10^−/−^* male mice fed either chow or high-fat diet at the indicated time periods after overnight fasting using the commercially available kit [45]. Around 20 µL of blood was collected from each mouse by tail vein bleeding for the analysis of plasma glucose. Glucose tolerance test (GTT) was performed after 14 weeks of chow and high-fat diet feeding in mice fasted overnight (~16 h) with full access to water. Mice were injected intraperitoneally with 2 mg of glucose in saline solution/g of body weight [46]. A tiny drop of blood (less than 10 µL) from the tail vein was used to measure blood glucose at the indicated times using a One-Touch basic glucometer (Bayer, Whippany, NJ, USA). For the insulin tolerance test (ITT), mice were fasted for 4 h prior to intraperitoneal injection of insulin (Novolin R, Novo Nordisk, Denmark; 0.75 U/kg body weight). Blood glucose levels were measured at the indicated time points before and after insulin injection [47].

### 4.5. Uptake and Secretion of Lipids by Primary Enterocytes

To study the lipid uptake, primary enterocytes from 12-week-old overnight-fasted *Abcc10^+/+^* and *Abcc10^−/−^* male mice (*n* = 3) were suspended in 4 mL of DMEM containing 0.5 μCi/mL of 3H-cholesterol or 14C-oleic acid and incubated at 37 °C [48,49]. After every 10 min, enterocyte samples from each condition were collected, washed, and centrifuged. Lipids were isolated from the cells to determine uptake of radiolabeled lipids. For the characterization of secreted lipoproteins, enterocytes were isolated from overnight-fasted mice and labeled for 1 h with 0.5 μCi/mL of 3H-cholesterol or 14C-oleic acid. Enterocytes were washed and incubated with fresh media containing 1.4 mM oleic acid containing micelles [48]. After 2 h, enterocytes were centrifuged, and supernatants were collected to measure secreted radiolabeled lipids in the media. Pellets were washed and lipids were isolated to determine the remaining cellular radiolabeled lipids.

### 4.6. Short-Term Lipid Absorption Studies

Age-matched male mice (3 per group) on a chow diet were fasted overnight and injected intraperitoneally with poloxamer 407 (P407, 30 mg/mouse). After 1 h, mice were gavaged with 0.5 μCi of either 3H-cholesterol or 14C-triolein with 0.2 mg of unlabeled cholesterol in 15 μL of olive oil [50]. After 2 h, collected plasma was used to measure radioactivity.

### 4.7. Hepatic Lipid Mobilization and Metabolic Studies

Hepatic cholesterol and triglyceride secretion rates were determined in vivo [50]. Mice (4 per group) were fasted for 16 h and injected intraperitoneally with P407 (30 mg/mouse). Blood samples were collected through tail vein bleeding before the injection of P407 and up to 3 h at indicated times for cholesterol and triglyceride determinations in the plasma.

### 4.8. Determination of MTP Activity

Small pieces (0.1 g) of liver or proximal small intestine (~1 cm) were homogenized in low-salt buffer (1 mM Tris-HCl, pH 7.6, 1 mM EGTA, and 1 mM MgCl_2_) and centrifuged, and supernatants were used for protein determination and MTP assay [51].

### 4.9. Western Blot Analysis

For the detection of proteins in liver and intestine, tissues were homogenized with RIPA buffer and were separated on 4–20% Mini-PROTEAN TGX precast protein gels (catalog #4561096) from BioRad (Hercules, CA, USA). Separated proteins were transferred to a PVDF membrane, blocked with 50 mM Tris, pH 7.6, 150 mM NaCl, 0.5% Tween 20, and 5% milk (TBS plus Tween 20), and probed with different primary antibodies (1:1000 dilution) overnight at 4 °C, followed by incubation with the secondary antibodies conjugated with peroxidase for 1 h at room temperature. The blots were developed with the Clarity Western ECL substrate (catalog #1705060) from BioRad (Hercules, CA, USA). The results were photographed with the ChemiDoc MP Imaging System from BioRad (Hercules, CA, USA). The band density of each protein was measured using the ImageJ 1.53k software (National Institutes of Health, Rockville, MD, USA).

### 4.10. mRNA Quantification

Total RNA from tissues was isolated using TRIzol^TM^. The purity of RNA was assessed by the A_260_/A_280_ ratio. RNA preparations with A_260_/A_280_ ratios more than 1.7 were used for cDNA synthesis. The first-strand cDNA was synthesized using the Omniscript RT kit. Each reaction of quantitative PCR was carried out in a volume of 20 µL, consisting of 10 µL of cDNA sample (1:100 dilution of the first strand cDNA sample) and 10 µL of PCR master mix solution containing 1X PCR buffer from the qPCR^TM^ core kit for SYBR Green I. The PCR was carried out by incubating the reaction mixture first for 10 min at 95 °C, followed by 40 cycles of 15 s incubations at 95 °C and 1 min at 60 °C in a QuantStudio^TM^ 6 Flex Real-Time PCR system (Applied Biosystems, Waltham, MA, USA). Data were analyzed using the ΔΔC_T_ method, according to the manufacturer’s instructions, and presented as arbitrary units that were normalized to *ArpP0* mRNA.

### 4.11. Mouse Atherosclerotic Lesion Measurement

Age-matched (12-week-old males) *Abcc10^−/^*, *Apoe^−/−^* double-knockout, and *Apoe^−/−^* mice were fed a Western-type diet for 12 weeks. The aortic arches were dissected, photographed, and quantified. Exposed aortas were stained with Oil Red O and an *en face* assay was performed [52].

### 4.12. Statistical Analysis

All data are presented as the mean ± S.D. The mean values of each group were analyzed via Student’s *t* test using GraphPad Prism software (version 5.0; GraphPad, San Diego, CA, USA). The results with *p* < 0.05 were considered statistically significant.

## Data Availability

The authors confirm that the data supporting the findings of this study are available within the article.

## References

[1] Hussain M.M., Shi J., Dreizen P. (2003). Microsomal triglyceride transfer protein and its role in apoB-lipoprotein assembly. J. Lipid Res..

[2] Iqbal J., Hussain M.M. (2009). Intestinal lipid absorption. Am. J. Physiol. Endocrinol. Metab..

[3] Packard C.J., Boren J., Taskinen M.R. (2020). Causes and Consequences of Hypertriglyceridemia. Front. Endocrinol..

[4] Nissen S.E., Nicholls S.J., Sipahi I., Libby P., Raichlen J.S., Ballantyne C.M., Davignon J., Erbel R., Fruchart J.C., Tardif J.C. (2006). Effect of very high-intensity statin therapy on regression of coronary atherosclerosis: The ASTEROID trial. JAMA.

[5] Boekholdt S.M., Hovingh G.K., Mora S., Arsenault B.J., Amarenco P., Pedersen T.R., LaRosa J.C., Waters D.D., DeMicco D.A., Simes R.J. (2014). Very low levels of atherogenic lipoproteins and the risk for cardiovascular events: A meta-analysis of statin trials. J. Am. Coll. Cardiol..

[6] Zimmet P., Alberti K.G., Shaw J. (2001). Global and societal implications of the diabetes epidemic. Nature.

[7] Taskinen M.R. (2003). Diabetic dyslipidaemia: From basic research to clinical practice. Diabetologia.

[8] Adiels M., Olofsson S.O., Taskinen M.R., Boren J. (2006). Diabetic dyslipidaemia. Curr. Opin. Lipidol..

[9] Ginsberg H.N., Zhang Y.L., Hernandez-Ono A. (2006). Metabolic syndrome: Focus on dyslipidemia. Obesity.

[10] Chan D.C., Watts G.F., Redgrave T.G., Mori T.A., Barrett P.H. (2002). Apolipoprotein B-100 kinetics in visceral obesity: Associations with plasma apolipoprotein C-III concentration. Metabolism.

[11] Veilleux A., Grenier E., Marceau P., Carpentier A.C., Richard D., Levy E. (2014). Intestinal lipid handling: Evidence and implication of insulin signaling abnormalities in human obese subjects. Arterioscler. Thromb. Vasc. Biol..

[12] Dassa E., Bouige P. (2001). The ABC of ABCS: A phylogenetic and functional classification of ABC systems in living organisms. Res. Microbiol..

[13] Kimura Y., Morita S.Y., Matsuo M., Ueda K. (2007). Mechanism of multidrug recognition by MDR1/ABCB1. Cancer Sci..

[14] Hirayama H., Kimura Y., Kioka N., Matsuo M., Ueda K. (2013). ATPase activity of human ABCG1 is stimulated by cholesterol and sphingomyelin. J. Lipid Res..

[15] Sharom F.J. (2008). ABC multidrug transporters: Structure, function and role in chemoresistance. Pharmacogenomics.

[16] Schmitz G., Langmann T. (2001). Structure, function and regulation of the ABC1 gene product. Curr. Opin. Lipidol..

[17] Francis G.A., Knopp R.H., Oram J.F. (1995). Defective removal of cellular cholesterol and phospholipids by apolipoprotein A-I in Tangier Disease. J. Clin. Investig..

[18] Iqbal J., Walsh M.T., Hammad S.M., Cuchel M., Tarugi P., Hegele R.A., Davidson N.O., Rader D.J., Klein R.L., Hussain M.M. (2015). Microsomal triglycerdie transfer protein transfers and determines plasma concentrations of ceramide and sphingomyelin but not glycosylceramide. J. Biol. Chem..

[19] Iqbal J., Suarez M.D., Yadav P.K., Walsh M.T., Li Y., Wu Y., Huang Z., James A.W., Escobar V., Mokbe A. (2022). ATP-binding cassette protein ABCA7 deficiency impairs sphingomyelin synthesis, cognitive discrimination, and synaptic plasticity in the entorhinal cortex. J. Biol. Chem..

[20] Raggers R.J., van Helvoort A., Evers R., van Meer G. (1999). The human multidrug resistance protein MRP1 translocates sphingolipid analogs across the plasma membrane. J. Cell Sci..

[21] Ishibashi Y., Kohyama-Koganeya A., Hirabayashi Y. (2013). New insights on glucosylated lipids: Metabolism and functions. Biochim. Biophys. Acta.

[22] Budani M., Auray-Blais C., Lingwood C. (2021). ATP-binding cassette transporters mediate differential biosynthesis of glycosphingolipid species. J. Lipid Res..

[23] Kathawala R.J., Wang Y.J., Ashby C.R., Chen Z.S. (2014). Recent advances regarding the role of ABC subfamily C member 10 (ABCC10) in the efflux of antitumor drugs. Chin. J. Cancer.

[24] Tarling E.J., de Aguiar Vallim T.Q., Edwards P.A. (2013). Role of ABC transporters in lipid transport and human disease. Trends Endocrinol. Metab..

[25] Westerterp M., Bochem A.E., Yvan-Charvet L., Murphy A.J., Wang N., Tall A.R. (2014). ATP-binding cassette transporters, atherosclerosis, and inflammation. Circ. Res..

[26] Pahnke J., Langer O., Krohn M. (2014). Alzheimer’s and ABC transporters—New opportunities for diagnostics and treatment. Neurobiol. Dis..

[27] Kotlyarov S., Kotlyarova A. (2021). Analysis of ABC Transporter Gene Expression in Atherosclerosis. Cardiogenetics.

[28] Surwit R.S., Kuhn C.M., Cochrane C., McCubbin J.A., Feinglos M.N. (1988). Diet-induced type II diabetes in C57BL/6J mice. Diabetes.

[29] Hariri N., Thibault L. (2010). High-fat diet-induced obesity in animal models. Nutr. Res. Rev..

[30] Bakillah A., Hussain M.M. (2016). Mice subjected to aP2-Cre mediated ablation of microsomal triglyceride transfer protein are resistant to high fat diet induced obesity. Nutr. Metab..

[31] Iqbal J., Dai K., Seimon T., Jungreis R., Oyadomari M., Kuriakose G., Ron D., Tabas I., Hussain M.M. (2008). IRE1beta inhibits chylomicron production by selectively degrading MTP mRNA. Cell Metab..

[32] Xie Y., Newberry E.P., Young S.G., Robine S., Hamilton R.L., Wong J.S., Luo J., Kennedy S., Davidson N.O. (2006). Compensatory increase in hepatic lipogenesis in mice with conditional intestine-specific Mttp deficiency. J. Biol. Chem..

[33] Kendrick J.S., Chan L., Higgins J.A. (2001). Superior role of apolipoprotein B48 over apolipoprotein B100 in chylomicron assembly and fat absorption: An investigation of apobec-1 knock-out and wild-type mice. Biochem. J..

[34] Tsuchida T., Fukuda S., Aoyama H., Taniuchi N., Ishihara T., Ohashi N., Sato H., Wakimoto K., Shiotani M., Oku A. (2012). MGAT2 deficiency ameliorates high-fat diet-induced obesity and insulin resistance by inhibiting intestinal fat absorption in mice. Lipids Health Dis..

[35] Yen C.L., Cheong M.L., Grueter C., Zhou P., Moriwaki J., Wong J.S., Hubbard B., Marmor S., Farese R.V. (2009). Deficiency of the intestinal enzyme acyl CoA:monoacylglycerol acyltransferase-2 protects mice from metabolic disorders induced by high-fat feeding. Nat. Med..

[36] Costa D.K., Huckestein B.R., Edmunds L.R., Petersen M.C., Nasiri A., Butrico G.M., Abulizi A., Harmon D.B., Lu C., Mantell B.S. (2016). Reduced intestinal lipid absorption and body weight-independent improvements in insulin sensitivity in high-fat diet-fed Park2 knockout mice. Am. J. Physiol. Endocrinol. Metab..

[37] Buhman K.K., Smith S.J., Stone S.J., Repa J.J., Wong J.S., Knapp F.F., Burri B.J., Hamilton R.L., Abumrad N.A., Farese R.V. (2002). DGAT1 is not essential for intestinal triacylglycerol absorption or chylomicron synthesis. J. Biol. Chem..

[38] Smith S.J., Cases S., Jensen D.R., Chen H.C., Sande E., Tow B., Sanan D.A., Raber J., Eckel R.H., Farese R.V. (2000). Obesity resistance and multiple mechanisms of triglyceride synthesis in mice lacking Dgat. Nat. Genet..

[39] Chen H.C., Ladha Z., Smith S.J., Farese R.V. (2003). Analysis of energy expenditure at different ambient temperatures in mice lacking DGAT1. Am. J. Physiol. Endocrinol. Metab..

[40] Iqbal J., Qarni A.A., Bakillah A. (2021). Diet-induced differential effects on plasma lipids secretion by the inositol-requiring transmembrane kinase/endoribonuclease 1alpha. Front. Biosci..

[41] Iqbal J., Parks J.S., Hussain M.M. (2013). Lipid absorption defects in intestine-specific microsomal triglyceride transfer protein and ATP-binding cassette transporter A1-deficient mice. J. Biol. Chem..

[42] Nelson R.H. (2013). Hyperlipidemia as a risk factor for cardiovascular disease. Prim. Care.

[43] Hopper-Borge E.A., Churchill T., Paulose C., Nicolas E., Jacobs J.D., Ngo O., Kuang Y., Grinberg A., Westphal H., Chen Z.S. (2011). Contribution of Abcc10 (Mrp7) to in vivo paclitaxel resistance as assessed in Abcc10(-/-) mice. Cancer Res..

[44] Iqbal J., Rudel L.L., Hussain M.M. (2008). Microsomal triglyceride transfer protein enhances cellular cholesteryl esterification by relieving product inhibition. J. Biol. Chem..

[45] Ayala J.E., Samuel V.T., Morton G.J., Obici S., Croniger C.M., Shulman G.I., Wasserman D.H., McGuinness O.P. (2010). Standard operating procedures for describing and performing metabolic tests of glucose homeostasis in mice. Dis. Model. Mech..

[46] Dinger K., Mohr J., Vohlen C., Hirani D., Hucklenbruch-Rother E., Ensenauer R., Dotsch J., Alejandre Alcazar M.A. (2018). Intraperitoneal Glucose Tolerance Test, Measurement of Lung Function, and Fixation of the Lung to Study the Impact of Obesity and Impaired Metabolism on Pulmonary Outcomes. J. Vis. Exp..

[47] Nagy C., Einwallner E. (2018). Study of In Vivo Glucose Metabolism in High-fat Diet-fed Mice Using Oral Glucose Tolerance Test (OGTT) and Insulin Tolerance Test (ITT). J. Vis. Exp..

[48] Iqbal J., Hussain M.M. (2005). Evidence for multiple complementary pathways for efficient cholesterol absorption in mice. J. Lipid Res..

[49] Iqbal J., Anwar K., Hussain M.M. (2003). Multiple, independently regulated pathways of cholesterol transport across the intestinal epithelial cells. J. Biol. Chem..

[50] Iqbal J., Queiroz J., Li Y., Jiang X.C., Ron D., Hussain M.M. (2012). Increased intestinal lipid absorption caused by Ire1beta deficiency contributes to hyperlipidemia and atherosclerosis in apolipoprotein E-deficient mice. Circ. Res..

[51] Athar H., Iqbal J., Jiang X.C., Hussain M.M. (2004). A simple, rapid, and sensitive fluorescence assay for microsomal triglyceride transfer protein. J. Lipid Res..

[52] Liu J., Huan C., Chakraborty M., Zhang H., Lu D., Kuo M.S., Cao G., Jiang X.C. (2009). Macrophage sphingomyelin synthase 2 deficiency decreases atherosclerosis in mice. Circ. Res..

